# Antiplatelet Resumption After Intracerebral Hemorrhage: A Systematic Review and Meta-Analysis

**DOI:** 10.3390/diagnostics15141780

**Published:** 2025-07-15

**Authors:** Sarah Yahya Alharthi, Sarah Abdulaziz Alsheikh, Dawood Salman Almousa, Saud Samer A. Alsedrah, Nouf Mohammed Alshammari, Mariam Mostafa Elsayed, Rahaf Ali Hamed AlShamrani, Mohammed Ahmed Yaslam Bellahwal, Abdulrahman Alnwiji, Raed A. Albar, Ayman M. A. Mohamed

**Affiliations:** 1 College of Medicine, Alfaisal University, Riyadh 11533, Saudi Arabia; syalharthi@alfaisal.edu (S.Y.A.); saralsheikh@alfaisal.edu (S.A.A.); dsalmousa@alfaisal.edu (D.S.A.); ssalsedrah@alfaisal.edu (S.S.A.A.); nalshammari@alfaisal.edu (N.M.A.); mbellahwal@alfaisal.edu (M.A.Y.B.); aalnwiji@alfaisal.edu (A.A.); ralbar@alfaisal.edu (R.A.A.); 2 College of Medicine, AlMaarefa University, P.O. Box 71666, Riyadh 11597, Saudi Arabia; 201220211@student.um.edu.sa (M.M.E.); 191220492@student.um.edu.sa (R.A.H.A.)

**Keywords:** ICH, antiplatelet, intracerebral hemorrhage, stroke

## Abstract

**Background:** Intracerebral hemorrhage management presents clinicians with a significant therapeutic challenge. Maintaining antiplatelet therapy potentially increases the risk of recurrent bleeding, while discontinuation heightens susceptibility to ischemic stroke, particularly during the critical first month after hemorrhage. In contemporary practice, physicians demonstrate considerable hesitancy regarding early antiplatelet reinitiation, complicated by the absence of clear evidence-based treatment guidelines. **Aim:** This meta-analysis assesses the safety of early antiplatelet resumption following ICH. **Methods**: We conducted a systematic review by searching Web of Science, Scopus, PubMed, and Cochrane Library from inception to April 2025. Articles were independently screened and data extracted by two reviewers who also assessed study quality. The inclusion criteria are enrollment of adults (≥18 years) with imaging-confirmed intracerebral hemorrhage surviving >24 h, comparing early vs. delayed or withheld antiplatelet therapy. Randomized trials underwent separate evaluation using Cochrane’s Risk of Bias. Statistical analysis was performed using R software (version 4.4.2), with categorical outcomes pooled as risk ratios (RRs) with 95% confidence intervals. Statistical significance was established at *p* < 0.05. The evidence is limited by the availability of few RCTs, variable antiplatelet regiments, male predominance, and other confounding factors. The review was registered in SFO. **Results:** Our meta-analysis included 10 studies (8 observational, 2 RCTs) with 5554 patients. Early antiplatelet therapy significantly reduced recurrent intracerebral hemorrhage by 46% (RR 0.54, 95% CI 0.37–0.78, *p* = 0.001). All-cause mortality showed a non-significant difference (RR 0.81, 95% CI 0.65–1.01, *p* = 0.06). No significant differences were found for ischemic stroke (RR 0.99, 95% CI 0.60–1.63, *p* = 0.96), major hemorrhagic events (RR 0.75, 95% CI 0.49–1.13, *p* = 0.17), or ischemic vascular outcomes (RR 0.71, 95% CI 0.49–1.02, *p* = 0.60). **Conclusions:** Our meta-analysis reveals that early antiplatelet therapy following intracerebral hemorrhage significantly reduces recurrent hemorrhagic events (46% reduction) without increasing major ischemic or hemorrhagic complications.

## 1. Introduction

Stroke constitutes a leading global mortality cause, with both hemorrhagic and ischemic variants demonstrating substantial long-term recurrence. Antiplatelet therapy preceding acute intracerebral hemorrhage correlates with increased hemorrhagic risk and unfavorable outcomes, necessitating routine antithrombotic discontinuation during acute management [1,2,3,4]. Beyond re-hemorrhage concerns, substantial ischemic cerebrovascular risk persists—3.0% annually and 2.8 per 100 person-years among non-atrial fibrillation patients, with dramatically elevated rates (27.3 per 100 person-years for ischemic stroke/systemic embolism and all-cause mortality) in atrial fibrillation cohorts [5,6,7]. Hemorrhage survivors experiencing subsequent arterial ischemic events face mortality rates reaching 21.7 per 100 person-years [8].

Antiplatelet resumption following intracerebral hemorrhage creates a significant clinical management paradox: administration potentially elevates re-hemorrhage risk, while abstention may increase ischemic event vulnerability. Contemporary evidence, including both observational research and the RESTART randomized trial, indicates that antiplatelet reinitiation carries modest re-hemorrhage risk that appears clinically acceptable when balanced against potential vascular protective benefits in occlusive disease prevention [9,10,11].

Antithrombotic reinitiation timing requires careful consideration of competing risks—recurrent hemorrhage versus ischemic complications. Evidence indicates that acute ischemic stroke risk peaks during the first month after hemorrhage [12]. Paradoxically, clinical practice data reveal that only 5% of patients receive antiplatelet agents within this critical 30-day window [13]. This substantial practice–evidence discordance exists within a guideline vacuum, as current recommendations provide no specific timing guidance for post-hemorrhagic antiplatelet resumption.

This evidence gap has resulted in an absence of well-established antithrombotic safety or efficacy among intracerebral hemorrhage survivors [14,15]. The cumulative evidence gap has hindered definitive guideline formulation, resulting in management inconsistencies. Comprehensive synthesis of existing evidence is fundamental for establishing evidence-based protocols in this clinically complex population. Our meta-analysis evaluated whether prompt antiplatelet reinitiation following spontaneous intracerebral hemorrhage favorably modulates re-hemorrhage incidence and major vascular event rates while preserving an acceptable hemorrhagic risk profile.

## 2. Materials and Methods

Our methodological approach adhered to established systematic review standards, incorporating both Cochrane Handbook principles and PRISMA (Preferred Reporting Items for Systematic Reviews and Meta-analyses) reporting guidelines [16,17]. We registered the protocol for this meta-analysis on OSF (https://doi.org/10.17605/OSF.IO/H5EJZ).

**Literature search**

We conducted a comprehensive literature search across multiple bibliographic databases (Web of Science, Cochrane CENTRAL, PubMed, Scopus) from their inception through April 2025. To maximize the retrieval of relevant studies, we supplemented electronic searching with manual reference list examination of eligible articles and pertinent meta-analyses. Our search protocol employed a carefully constructed combination of relevant keywords, as shown in Supplementary Table S1.

**Eligibility criteria**

Two independent reviewers assessed reference relevance using predefined eligibility criteria. Studies qualified for inclusion when meeting the following requirements: enrollment of adult subjects (≥18 years) with imaging-confirmed intracerebral hemorrhage who survived beyond 24 h, and a comparative analysis between early antiplatelet administration protocols versus delayed or withheld antiplatelet therapy. All participants had been receiving antiplatelet agents for vascular disease prevention when their hemorrhage occurred, subsequently discontinuing this treatment. Both observational studies and randomized control trials were included.

Exclusion criteria comprised studies with patients whose bleeding resulted from traumatic injury, hemorrhagic conversion of ischemic stroke, or isolated intracranial bleeding without parenchymal involvement. Additionally, those continuing antithrombotic therapy after hemorrhage were deemed ineligible for participation.

**Data collection**

Data were systematically collected using a standardized extraction form. For each study, we documented the identification parameters, methodological characteristics (arms, sample size, design, setting, follow-up duration, hemorrhage location), intervention details (type, dosage, duration, timing), demographic profile (mean age with standard deviation, gender distribution), comorbidity prevalence (diabetes, hypertension, smoking status, coronary pathology, prior cerebrovascular events, atrial fibrillation), hemorrhage classification (intracerebral, subarachnoid, subdural), antihypertensive medication use, eligibility requirements, and primary conclusions.

**Quality assessment**

For observational research, two reviewers independently applied the Newcastle–Ottawa Scale, which examines bias across three domains: Selection (population characteristics, control selection, case definition; maximum 4 points); Comparability (confounder adjustment including gestational age and maternal comorbidities; maximum 2 points); and Exposure (measurement standardization and blinding; maximum 3 points). This 9-point scale correlates higher scores with methodological strength [18]. Randomized trials underwent separate evaluation using Cochrane’s Risk of Bias Tool 2, examining five key domains: randomization integrity, intervention fidelity, outcome completeness, measurement objectivity, and reporting comprehensiveness. These assessments yielded categorical classifications (low-risk, some concerns, high-risk) [19]. Evaluator discrepancies underwent resolution through deliberative study.

**Data synthesis**

Statistical analyses for this meta-analysis were conducted using R software version 4.4.2, with categorical outcomes systematically pooled as risk ratios (RRs) accompanied by their corresponding 95% confidence intervals to ensure statistical precision. To rigorously evaluate between-study heterogeneity, both I^2^ statistics and chi-square testing were employed, with specific predetermined thresholds guiding interpretation—I^2^ values ≥ 50% were defined as indicative of substantial heterogeneity, while chi-square *p*-values exceeding 0.10 signaled significant statistical variation across studies. Throughout all the analyses, statistical significance was consistently established at the conventional threshold of *p* < 0.05. The methodological robustness and reliability of the findings underwent comprehensive verification through two complementary approaches: leave-one-out sensitivity analysis, which assessed the influence of individual studies on overall results, and Galbraith plot visualization techniques, which provided graphical representation of potential outliers and between-study relationships.

## 3. Results

**Literature Search Results**

**Figure 1:**
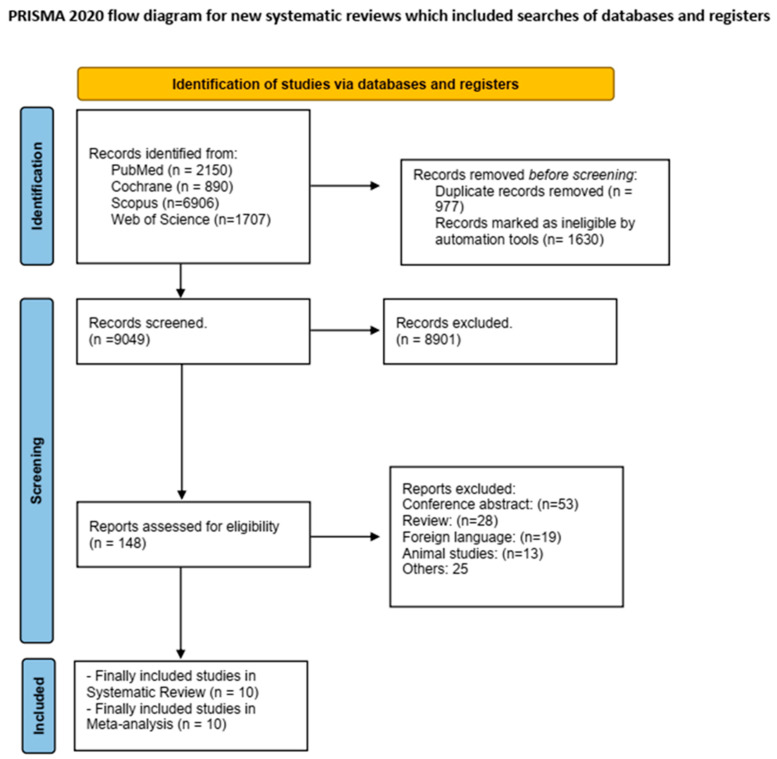
PRISMA Flow Diagram for Studies Selection Process.

A comprehensive literature search was conducted across multiple electronic databases, resulting in the identification of 11,653 records. Prior to screening, 2607 records were removed, comprising 977 duplicate records and 1630 records marked as ineligible by automation tools. The remaining 9049 records underwent initial screening, of which 8901 were excluded based on the title and abstract review. Of the 148 reports assessed for full-text eligibility, 138 were excluded. Ultimately, 10 studies met all the inclusion criteria and were incorporated into both the systematic review and meta-analysis [10,13,20,21,22,23,24,25,26,27]. Figure 1 shows the PRISMA flow diagram selection process.

**Included Study Characteristics**

Our meta-analysis included ten studies, comprising eight observational studies (both retrospective and prospective cohorts) and two randomized trials (RESTART Trial 2019 [10] and Liu 2024 [26]). These studies were geographically diverse, spanning China (3), the United Kingdom (2), South Korea (2), the United States (1), Taiwan (1), and Scotland (1). The follow-up duration across studies ranged considerably from 3 months to 6.4 years, with a median of approximately 2 years. The analysis encompassed a total of 5554 patients, with 2446 receiving early antiplatelet therapy and 3108 serving as controls. The patient population was predominantly male (47.5–73% across studies), with a mean age ranging from 58.7 to 76.0 years.

Regarding intracerebral hemorrhage (ICH) location, reporting varied between studies, with lobar hemorrhages accounting for 29.6–65.6% of cases and deep/non-lobar hemorrhages representing 33.3–65.6% when reported. Infratentorial hemorrhages constituted 10–13.6% in studies that documented this location. Comorbidity profiles revealed a high prevalence of hypertension (49.7–94.4%) and diabetes mellitus (13.8–47.8%) across the study populations. Other comorbidities showed considerable variability, including previous stroke/transient ischemic attack (3.3–67%), coronary artery disease (2.9–83.5%), and atrial fibrillation (2.9–25.2% in reporting studies). The antiplatelet interventions primarily involved aspirin (75–100 mg daily), though some studies also examined clopidogrel, cilostazol, ticagrelor, and dipyridamole, administered as either single or dual antiplatelet therapy. The timing of antiplatelet initiation varied significantly, ranging from as early as 3 days after surgery in Liu 2024 [26] to as late as 1-year after ICH, with most studies examining a timeframe of 30 days to 6 months after the hemorrhagic event. The baseline characteristics and a summary of the included studies are summarized in Supplementary Table S2.

**Quality Assessment**

For cohort studies, seven of the eight cohort studies demonstrated high quality with an appropriate selection of exposed and non-exposed cohorts, adequate ascertainment of exposure, and sufficient follow-up periods. These studies also showed strong comparability with adjustments for age and comorbidities. Only one cohort study (Moon 2021 [13]) was assessed as being of poor quality, primarily due to limitations in the outcome assessment and follow-up. For randomized controlled studies, both the E-start trial (Liu 2024) [26] and the RESTART Trial (2019) [10] exhibited low risk of bias across all domains. The detailed judgment is illustrated in Supplementary Table S3 and Supplementary Figure S1.

**Outcomes**

**Recurrent ICH**

**Figure 2:**
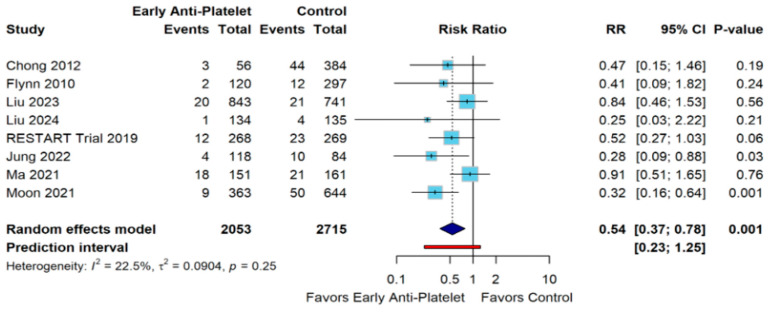
Forest plot for Recurrent ICH outcome [10,13,21,22,24,25,26,27].

Our meta-analysis compared early antiplatelet therapy versus control across 8 studies, with a total of 4768 participants (2053 in the early antiplatelet group and 2715 in the control group). The random effects model demonstrated that early antiplatelet therapy was associated with a statistically significant 46% reduction in events compared to control (RR 0.54, 95% CI 0.37–0.78, *p* = 0.001). Heterogeneity among studies was low to moderate (I^2^ = 22.5%, τ^2^ = 0.0904, *p* = 0.25) (Figure 2).

The leave-one-out sensitivity analysis demonstrated robust and consistent findings across all iterations of the meta-analysis. When systematically excluding each individual study, the pooled risk ratio remained statistically significant in all iterations, ranging from 0.47 (95% CI 0.32–0.70, *p* = 0.0002) when omitting Ma 2021 [27] to 0.63 (95% CI 0.45–0.88, *p* = 0.0077) when omitting Moon 2021 [13]. Notably, omitting Liu 2023 [25] produced the most favorable effect estimate (RR = 0.48, 95% CI 0.32–0.73, *p* = 0.0007), while omitting Moon 2021 [13] yielded the most conservative estimate. The consistency of statistical significance across all iterations, with *p*-values ranging from 0.0002 to 0.0077, confirms that no single study disproportionately influenced the overall findings (Supplementary Figure S2).

The Galbraith plot demonstrates the relationship between precision (1/SE) and standardized effect (z-score) across the eight included studies. Six studies (Liu 2024, Flynn 2010, Chong 2012, RESTART Trial 2019, Ma 2021, and Liu 2023) [10,21,22,25,26,27] fell within the 95% confidence bands, indicating consistency with the overall effect estimate. Two studies (Jung 2022 and Moon 2021) [13,24] appeared as statistical outliers (*p* < 0.05), positioned outside the confidence bands and represented by red dots, suggesting they contributed disproportionately to the observed treatment effect. The plot reveals a pattern where studies with higher precision (positioned further right on the x-axis) tended to report more pronounced treatment effects, particularly Moon 2021 [13], which showed the strongest standardized effect. The central regression line demonstrates the weighted relationship between precision and effect size, with most studies clustering around this line, supporting the overall homogeneity of the meta-analysis findings (Supplementary Figure S3).

**All-Cause Mortality**

**Figure 3:**
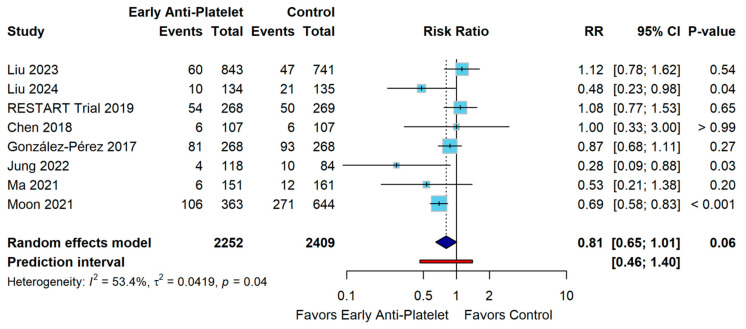
Forest Plot for All-Cause Mortality Outcomes [10,13,20,23,24,25,26,27].

Mortality was evaluated across 8 studies comprising 4661 participants (2252 in the early antiplatelet group and 2409 in the control group). The pooled analysis yielded a risk ratio of 0.81 (95% CI 0.65–1.01, *p* = 0.06), showing no significant difference between the two groups compared. Moderate to substantial heterogeneity was observed (I^2^ = 53.4%, τ^2^ = 0.0419, *p* = 0.04) (Figure 3).

When systematically excluding individual studies, the pooled risk ratio ranged from 0.76 (95% CI 0.60–0.96, *p* = 0.0205) when omitting Liu 2023 [25] to 0.85 (95% CI 0.66–1.10, *p* = 0.2080) when omitting Moon 2021 [13]. Only two iterations achieved statistical significance: omitting Liu 2023 [25] (*p* = 0.0205) and omitting RESTART Trial 2019 [10] (RR = 0.76, 95% CI 0.59–0.97, *p* = 0.0256) (Supplementary Figure S4).

Five studies (Chen 2018, Ma 2021, Liu 2023, RESTART Trial 2019, and González-Pérez 2017) [10,20,23,25,27] fell within the 95% confidence bands, indicating consistency with the overall effect estimate. Three studies (Liu 2024, Jung 2022, and Moon 2021) [13,24,26], represented by red dots, appeared as statistical outliers (*p* < 0.05) positioned outside the confidence bands, suggesting that they contributed disproportionately to the observed treatment effect. Studies with lower precision generally showed more variable effect estimates. This visual assessment complements the moderate heterogeneity statistics observed in the primary analysis and identifies Moon 2021, Jung 2022, and Liu 2024 [13,24,26] as key drivers of the treatment effect. The distribution pattern suggests that methodological differences or clinical heterogeneity among these studies may explain the variability in observed treatment effects, with the most precise study (Moon 2021 [13]) showing the most pronounced benefit of early antiplatelet therapy (Supplementary Figure S5).

**Ischemic Stroke**

**Figure 4:**
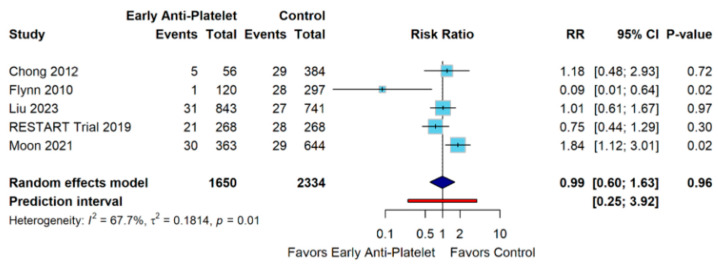
Forest Plot for Ischemic Stroke Outcome [10,13,21,22,25].

This meta-analysis evaluated early antiplatelet therapy versus control across 5 studies (Chong 2012, Flynn 2010, Liu 2023, RESTART Trial 2019, and Moon 2021) [10,13,21,22,25] comprising 3984 participants (1650 in the experimental group and 2334 in the control group). The random effects model yielded a risk ratio of 0.99 (95% CI 0.60–1.63, *p* = 0.96), indicating no statistically significant difference between early antiplatelet therapy and control. Substantial heterogeneity was observed (I^2^ = 67.7%, τ^2^ = 0.1814, *p* = 0.01), reflecting the inconsistent treatment effects across studies (Figure 4).

**Secondary Outcomes**

**Figure 5:**
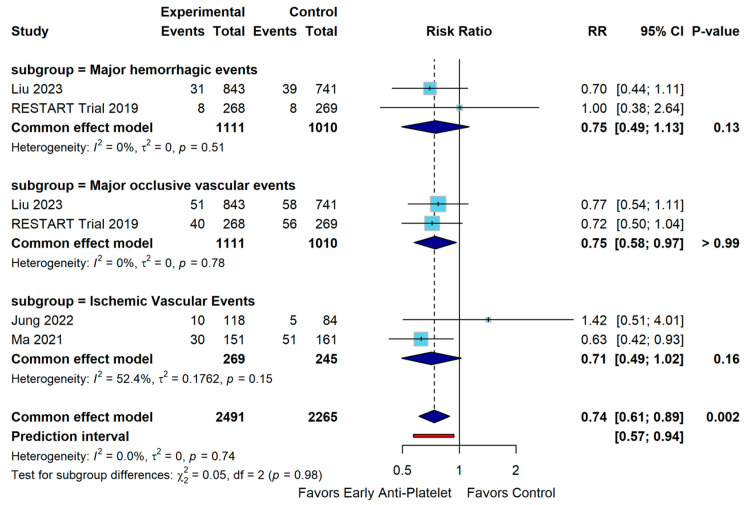
Forest Plot for Secondary Outcomes [10,24,25,27].

Analysis of early antiplatelet therapy revealed no statistically significant reduction in major hemorrhagic events across two studies involving 2121 subjects (RR 0.75, 95% CI 0.49–1.13, *p* = 0.17). Examination of ischemic vascular outcomes in a smaller subset (514 participants, 2 studies) likewise showed a non-significant difference (RR 0.71, 95% CI 0.49–1.02, *p* = 0.60). However, early antiplatelet therapy demonstrated a statistically significant difference for major occlusive vascular events in the same population (RR 0.75, 95% CI 0.58–0.97, *p* = 0.03) (Figure 5).

## 4. Discussion

Our meta-analysis, comprising ten studies (n = 5554), demonstrates that early antiplatelet therapy is associated with a statistically significant 46% reduction in recurrent intracerebral hemorrhage (RR 0.54, 95% CI 0.37–0.78). The observed effect remained consistent throughout the sensitivity analyses, confirming the robustness of these findings. Notably, early antiplatelet therapy exhibited no statistically significant effect on all-cause mortality, ischemic stroke incidence, major hemorrhagic events, or ischemic vascular outcomes. These findings contradict conventional clinical concerns and suggest that early antiplatelet administration following intracerebral hemorrhage may provide significant prophylaxis against recurrent hemorrhagic events without increasing the risk of thromboembolic complications. This evidence challenges existing paradigms in post-hemorrhagic stroke management protocols and warrants consideration in clinical practice guidelines.

Concerns regarding antiplatelet-associated hemostatic impairment prompted the exclusion of patients with a significant bleeding history from definitive antiplatelet randomized controlled trials. This evidentiary gap has fostered clinical uncertainty regarding post-hemorrhagic antiplatelet therapy, particularly given recurrence concerns and specific apprehension regarding lobar hemorrhages with cerebral amyloid angiopathy markers—a cohort demonstrating elevated re-hemorrhage risk. Consequently, only approximately 15% of hemorrhage survivors receive antiplatelets at discharge [13,21,22,28,29,30,31]. Counterbalancing arguments include established major adverse cardiovascular event reduction with antiplatelet therapy, substantial post-hemorrhagic cardiovascular risk profiles, predominance of ischemic over hemorrhagic complications following intracerebral hemorrhage, and potentially modest re-hemorrhage risk increases with antiplatelet use—suggesting a potential net benefit [32]. Nevertheless, concerns regarding amplified recurrence probability and hemorrhage severity create persistent uncertainty regarding ultimate mortality and disability outcomes, despite apparent ischemic event reduction.

Previous investigations into antiplatelet therapy following spontaneous intracerebral hemorrhage yielded inconsistent outcomes, notably excluding neurosurgical populations. While a large retrospective analysis demonstrated comparable rates of ischemic and hemorrhagic events between early (≤30 days) and delayed (31–365 days) antiplatelet initiation, the RESTART Trial confirmed safety but not efficacy for ischemic prevention [10,25]. Conversely, POISE-2 found no cardiovascular benefit from early postoperative acetylsalicylic acid administration in non-cardiac surgery [33]. Our investigation demonstrated a 46% risk reduction for recurrent ICH in favor of early antiplatelet initiation.

Meta-analysis of observational research encompassing various intracranial hemorrhage subtypes demonstrated a reduced ischemic event risk (RR 0.61, 95% CI 0.48–0.79) without a significant hemorrhagic complication increase (RR 0.84, 95% CI 0.47–1.51) following antiplatelet resumption versus avoidance [34]. Subsequent small-scale studies specifically examining intracerebral hemorrhage populations yielded comparable findings and suggested relative safety of early antiplatelet reinitiation within 30 days after hemorrhage [13,21,22,28,29,30,31]. However, one investigation identified that antiplatelet use following lobar hemorrhage was independently associated with recurrence in multivariate modeling [30]. Despite these encouraging observations, inherent methodological limitations—particularly selection bias and indication-based confounding—necessitate verification through randomized controlled trials before definitive clinical implementation.

A 2021 Cochrane systematic review identified six randomized controlled trials (three published, three unpublished) examining antithrombotic therapy following intracerebral hemorrhage [35]. Supplementary evidence emerged from two major ischemic stroke studies containing 773 participants inadvertently administered aspirin following hemorrhagic events (ultimately identified through subsequent imaging) [10,36]. They yielded reassuring short-term safety data (odds ratio 1.02, 95% CI 0.58–1.79), suggesting that aspirin administration for approximately two days did not significantly elevate recurrence risk—this is particularly noteworthy given that administration occurred during the period of maximal vulnerability to hematoma expansion and re-hemorrhage.

An E-start trial, which specifically enrolled subjects without rebleeding during the initial 72 postoperative hours, demonstrated intracranial hemorrhagic events in less than 1% of early antiplatelet recipients [26]. Their comparative analysis revealed no statistically significant between-group differences in hemorrhagic risk, moderate-to-severe bleeding, or symptomatic intracranial hemorrhage, though the limited event frequency precludes definitive safety conclusions [26]. These findings, aligned with RESTART Trial outcomes and related research, suggest the relative safety of early antiplatelet initiation following surgical intervention for spontaneous intracerebral hemorrhage [10]. Importantly, E-start protocol incorporated comprehensive neurological assessment and radiographic verification prior to antiplatelet administration, preventing inappropriate treatment of unstable patients.

The RESTART Trial represents the sole published randomized controlled trial examining long-term antiplatelet resumption following intracerebral hemorrhage [10,11,37]. This UK-based pilot study enrolled 537 adults who experienced hemorrhage while receiving antithrombotic therapy, randomizing them to either resume or avoid antiplatelet agents. Primary findings revealed that antiplatelet resumption was unlikely to increase the re-hemorrhage risk (4% versus 9%; adjusted hazard ratio 0.51, 95% CI 0.25–1.03, *p* = 0.060), suggesting potentially greater safety than anticipated based on secondary prevention data in non-hemorrhage populations. Major adverse cardiovascular event reduction (initial adjusted hazard ratio 0.65, 95% CI 0.44–0.95; extended follow-up adjusted hazard ratio 0.79, 95% CI 0.58–1.09) paralleled established secondary prevention benefits. Notably, subgroup analyses demonstrated consistent treatment effects regardless of hemorrhage location, timing, cardiac rhythm, or neuroimaging biomarkers [10,11,37]. Despite these encouraging findings, important limitations included the specific population (antithrombotic-associated hemorrhage with limited representation of hemorrhage survivors without prior cardiovascular events), predominantly UK-based recruitment restricting generalizability to diverse populations, open-label design, insufficient power for definitive conclusions or subgroup heterogeneity detection, effect attenuation during extended follow-up, and cardiovascular event reduction being a secondary rather than primary outcome [10,11,37].

Following RESTART, international guidelines have adopted cautiously permissive positions regarding antiplatelet therapy after hemorrhage. Canadian, Chinese, American, and British/Irish guidelines now suggest that antiplatelet resumption “may be reasonable” or “can be considered” for patients with pre-existing indications [38,39,40,41]. However, these recommendations universally carry B-level evidence grading, reflecting limited evidential strength. Chinese guidance specifically mentions potential early resumption (within days of onset) while acknowledging timing uncertainty. British/Irish guidelines emphasize individualized risk assessment balancing recurrence against thromboembolic hazards. Notably, all recommendations encourage clinical trial participation where feasible. To establish definitive A-level evidence addressing RESTART’s limitations, recruitment into an adequately powered confirmatory trial remains essential—excepting cases with compelling indications for specific antithrombotic approaches or where participation in alternative trials (e.g., anticoagulation or left atrial appendage occlusion for atrial fibrillation) takes precedence [38,39,40,41].

Regarding future research implications, a properly dimensioned confirmatory trial remains essential to establish treatment effects conclusively. Several knowledge gaps perpetuate guideline uncertainty: verification of net clinical benefit (mortality, disability, cognitive outcomes), effects across clinical and radiographic subgroups, and confirmation of RESTART’s primary safety observations. While RESTART examined high-risk populations with established vascular disease, subsequent investigation should encompass broader demographic profiles, including hemorrhage survivors without prior cardiovascular conditions. Current evidence demonstrates limited generalizability across ethnicities, healthcare systems, and resource environments. The predominance of male participants (approximately two-thirds) necessitates improved gender representation in future trials. Additional questions persist regarding optimal intervention timing relative to hemorrhage onset and comparative efficacy of specific antiplatelet regimens (monotherapy versus dual therapy, agent selection).

To our knowledge, this meta-analysis represents the most comprehensive synthesis to date on this topic, incorporating data from ten studies with a total of 5554 patients, including two high-quality randomized controlled trials (RCTs). The large, pooled sample size enhances statistical power, reduces the risk of type II error, and strengthens the robustness of our findings. We conducted rigorous sensitivity analyses, including leave-one-out analysis and Galbraith’s plot, to explore and clarify sources of heterogeneity. Most of the included studies were of high methodological quality. Nevertheless, limitations exist. The evidence base remains dominated by observational studies, with only two RCTs available, underscoring the need for larger, high-quality RCTs with standardized study designs and baseline characteristics. Variation in antiplatelet regimens and dosing, as well as inconsistencies in control group management (e.g., delayed or absent antiplatelet initiation), contribute to residual confounding. Important variables such as intracranial hemorrhage size and antiplatelet initiation protocols were often inadequately controlled. Additionally, the predominance of male participants (~two-thirds) limits gender generalizability, and current findings may not be fully applicable across diverse ethnic groups, healthcare settings, or resource-limited environments. Future studies should also address unresolved questions regarding the optimal timing and type of antiplatelet therapy (e.g., monotherapy vs. dual therapy, choice of agent), control confounders, and standardize these parameters to enable more definitive conclusions.

## 5. Conclusions

Our meta-analysis reveals that early antiplatelet therapy following intracerebral hemorrhage significantly reduces recurrent hemorrhagic events (46% reduction) without increasing major ischemic or hemorrhagic complications. This challenges conventional management approaches in post-hemorrhagic stroke care. Future research should prioritize large-scale, standardized RCTs investigating optimal timing, agent selection (monotherapy vs. dual therapy), diverse participant representation, and dosing strategies to help clinicians in individualizing antiplatelet resumption decisions based on each patient’s unique risk–benefit profile, considering factors such as hemorrhage characteristics and cardiovascular risk. Until more definitive evidence emerges, clinicians should cautiously consider early antiplatelet therapy in select patients following intracerebral hemorrhage, potentially revising current clinical practice guidelines.

## Data Availability

The original contributions presented in this study are included in the article/Supplementary Materials. Further inquiries can be directed to the corresponding author.

## Supplementary Materials

The following supporting information can be downloaded at: https://www.mdpi.com/article/10.3390/diagnostics15141780/s1, Figure S1: Risk of bias assessment using ROB-2 tool; Figure S2: Leave-One Out Analysis of Recurrent ICH Outcome; Figure S3: Galbraith Plot of Recurrent ICH Outcome; Figure S4: Leave-One Out Analysis of All-Cause Mortality Outcome; Figure S5: Galbraith Plot of All-Cause Mortality Outcome; Table S1: Search strategy for each database; Table S2: Baseline Characteristics and Summary of the Included Studies; Table S3: Detailed Judgment for Cohort Studies Based on Newcastle–Ottawa Scale (NOS). Reference [42] is cited in the supplementary materials.
