# Supplementary material for: Antiplatelet Resumption After Intracerebral Hemorrhage: A Systematic Review and Meta-Analysis
Source: Diagnostics (Basel). 2025 Jul 15;15(14):1780. doi: 10.3390/diagnostics15141780 (PMC12293368; doi:10.3390/diagnostics15141780)
Supplement: Supplementary file 1 [file diagnostics-15-01780-s001.zip › Supplemental Tables.docx]

**Supplementary Table 1. Search strategy for each database.**

| Results | Filter | Search query | Database |
| --- | --- | --- | --- |
| 2150 | All | ("antiplatelet therapy" OR "aspirin" OR "clopidogrel" OR "ticlopidine" OR "cilostazol" OR "dipyridamole" OR "antiplatelet withdrawal") AND ("intracranial hemorrhage" OR "cerebral hemorrhage" OR "brain hemorrhage" OR "spontaneous intracerebral hemorrhage" OR "ICH") | PubMed |
| 892 | All | ("antiplatelet therapy" OR "aspirin" OR "clopidogrel" OR "ticlopidine" OR "cilostazol" OR "dipyridamole" OR "no antiplatelet therapy" OR "antiplatelet withdrawal") AND ("intracranial hemorrhage" OR "cerebral hemorrhage" OR "brain hemorrhage" OR "spontaneous intracerebral hemorrhage" OR "ICH") | Cochrane |
| 1707 | All | ("antiplatelet therapy" OR "aspirin" OR "clopidogrel" OR "ticlopidine" OR "cilostazol" OR "dipyridamole" OR "no antiplatelet therapy" OR "antiplatelet withdrawal") AND ("intracranial hemorrhage" OR "cerebral hemorrhage" OR "brain hemorrhage" OR "spontaneous intracerebral hemorrhage" OR "ICH") | WOS |
| 6907 | Title, abstract, keywords | TITLE-ABS-KEY ( ( "antiplatelet therapy" OR "aspirin" OR "clopidogrel" OR "ticlopidine" OR "cilostazol" OR "dipyridamole" OR "no antiplatelet therapy" OR "antiplatelet withdrawal" ) AND ( "intracranial hemorrhage" OR "cerebral hemorrhage" OR "brain hemorrhage" OR "spontaneous intracerebral hemorrhage" OR "ICH" ) ) AND PUBYEAR > 1999 AND PUBYEAR < 2026 AND ( LIMIT-TO ( DOCTYPE , "ar" ) ) AND ( LIMIT-TO ( EXACTKEYWORD , "Human" ) ) AND ( LIMIT-TO ( LANGUAGE , "English" ) ) | Scopus |
| 11656 |  |  | Total |

Search strategy 01/04/2025

**Supplementary Table 3.** **Detailed Judgment for Cohort Studies Based on New-Castle Ottawa Scale (NOS)**

|  |  | **Cohort studies** |  |  |  |  |  |  |  |  |
| --- | --- | --- | --- | --- | --- | --- | --- | --- | --- | --- |
|  |  | **Selection** |  |  |  | **Comparability** | **Outcome** |  |  | **Quality Score** |
| **ID** | **Comparability:**  **Adjusted for:** 1) Age 2) Comorbidity | **Representativeness of the exposed cohort** | **Selection of the non-exposed cohort** | **Ascertainment of exposure** | **Demonstration of the outcome of interest was not present at start of study** | **Comparability of cohorts on the basis of the design or analysis** | **Assessment of outcome** | **Was follow-up long enough for outcomes to occur** | **Adequacy of follow up of cohorts** |  |
| Chong 2011 |  |  | ***** | ***** | ***** | ****** | ***** | ***** | ***** | **high quality** |
| Flynn 2010 |  |  | ***** | ***** | ***** | ****** | ***** | ***** | ***** | **high quality** |
| Liu 2023 |  | ***** | ***** | ***** | ***** | ****** | ***** | ***** | ***** | **high quality** |
| Chen 2018 |  | ***** | ***** | ***** | ***** | ***** | ***** | ***** | ***** | **high quality** |
| González-Pérez 2017 |  |  | ***** | ***** | ***** | ***** | ***** | ***** | ***** | **high quality** |
| Jung 2022 |  |  | ***** | ***** | ***** | ****** | ***** | ***** | ***** | **high quality** |
| Ma 2021 |  |  | ***** | ***** | ***** | ***** | ***** | ***** | ***** | **high quality** |
| Moon 2021 |  |  | ***** | ***** | ***** |  | ***** | ***** | ***** | **poor quality** |

**Supplementary Figure 1.** Risk of bias assessment using ROB-2 tool.


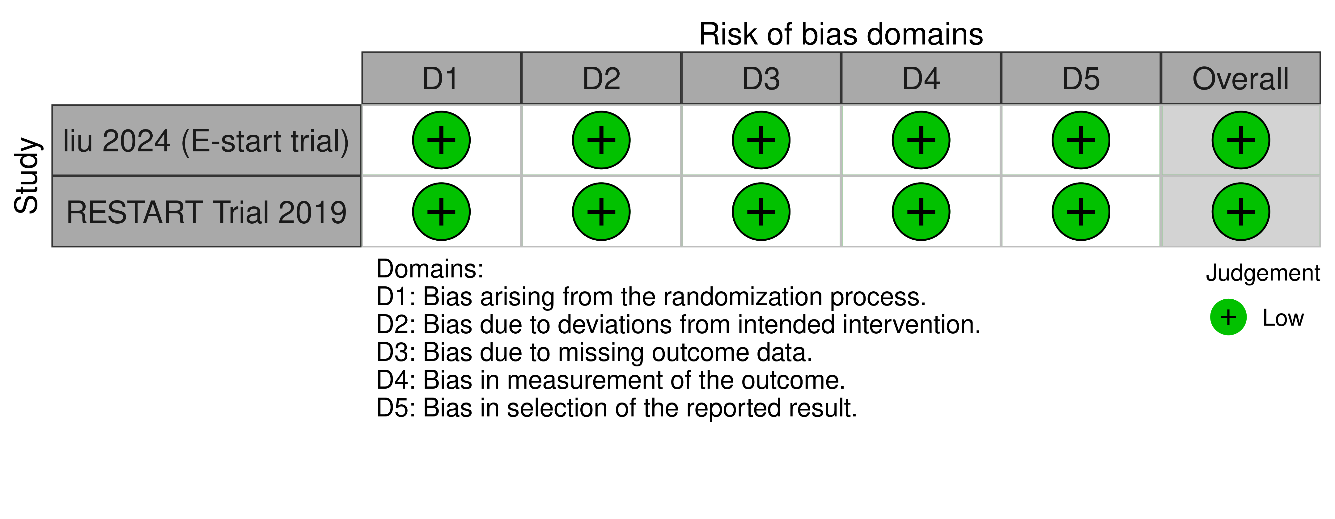


**Supplementary Figure 2: Leave-One Out Analysis of Recurrent ICH Outcome**


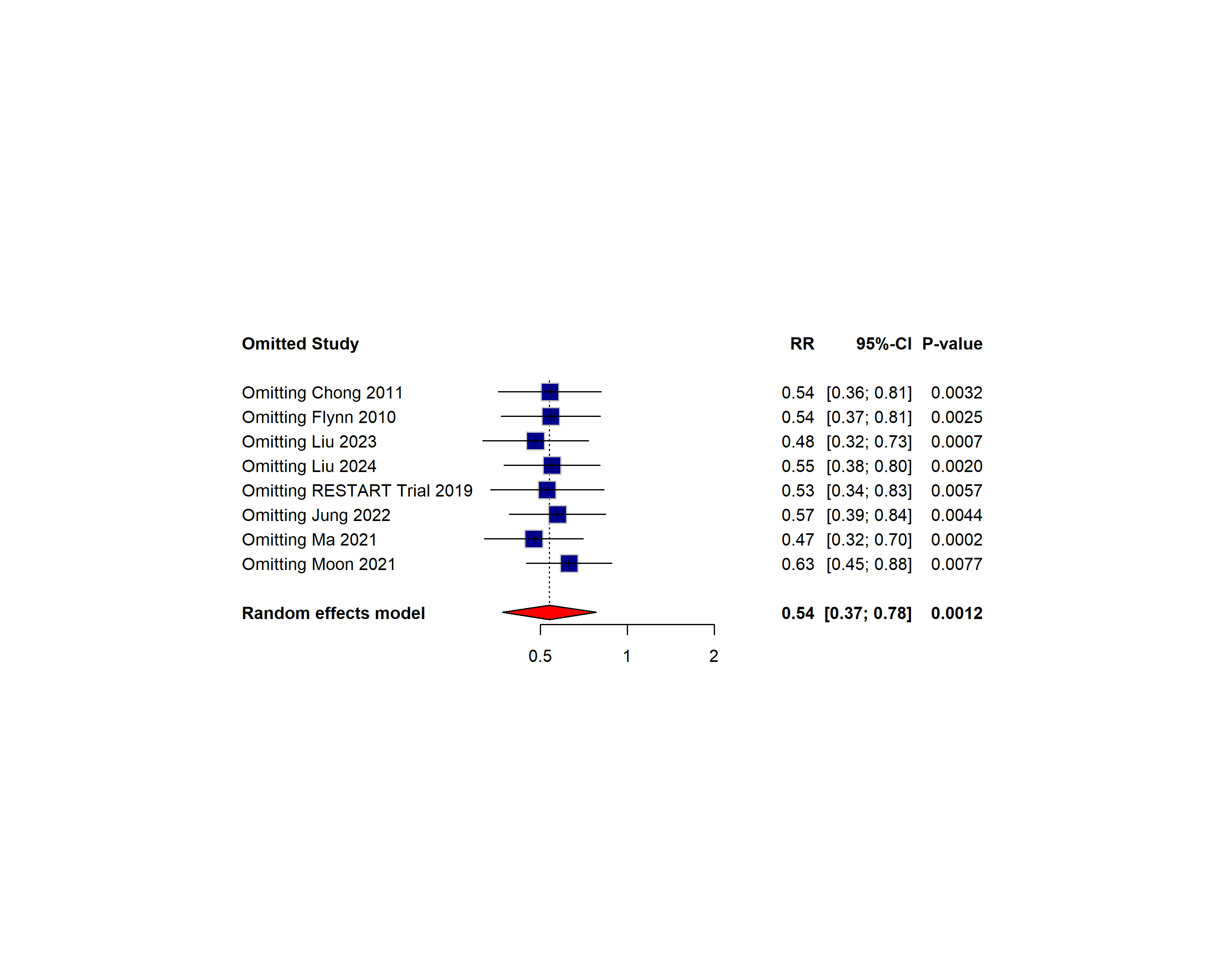


**Supplementary Figure 3. Galbraith Plot of Recurrent ICH Outcome**


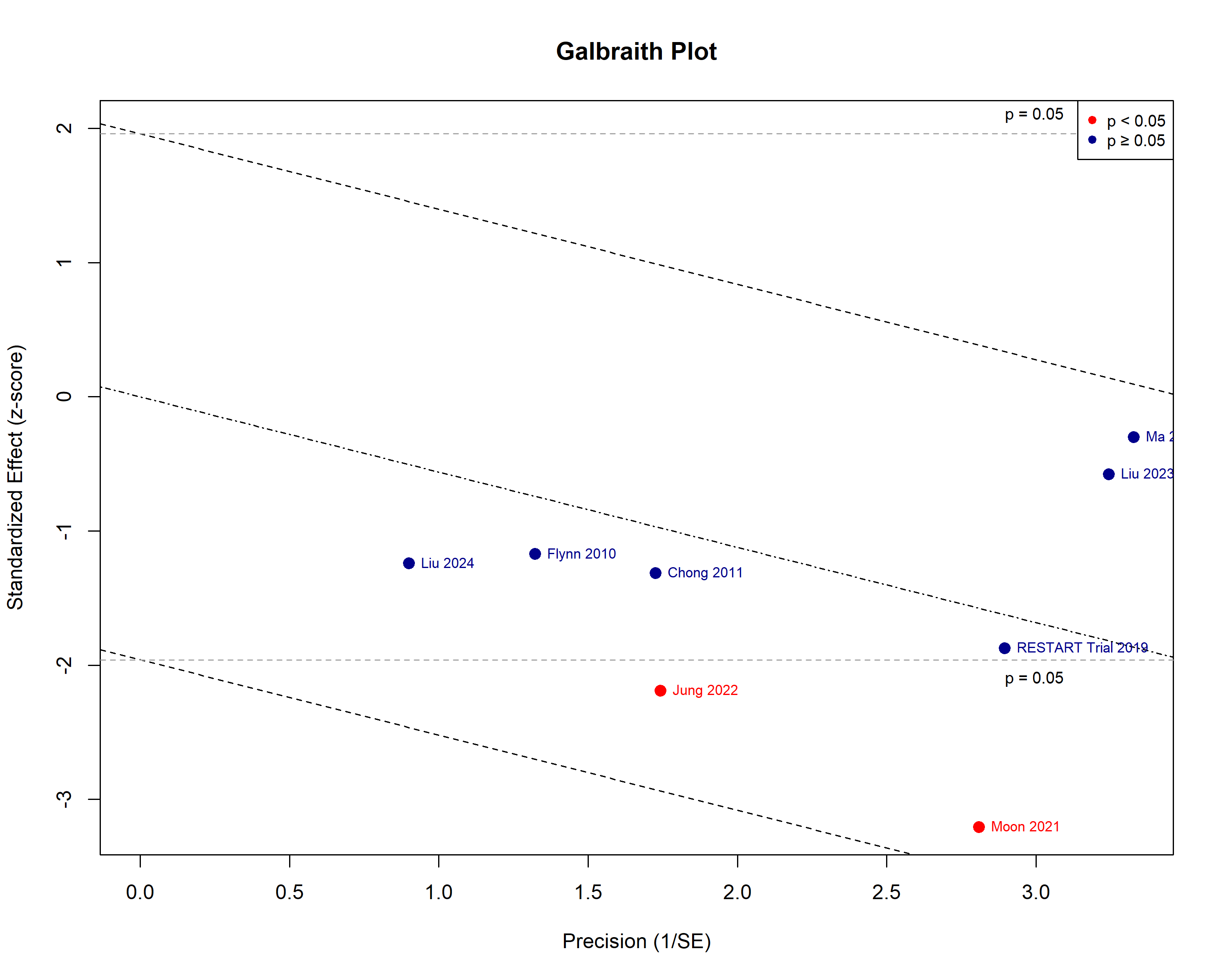


**Supplementary Figure 4. Leave-One Out Analysis of All-Cause Mortality Outcome**


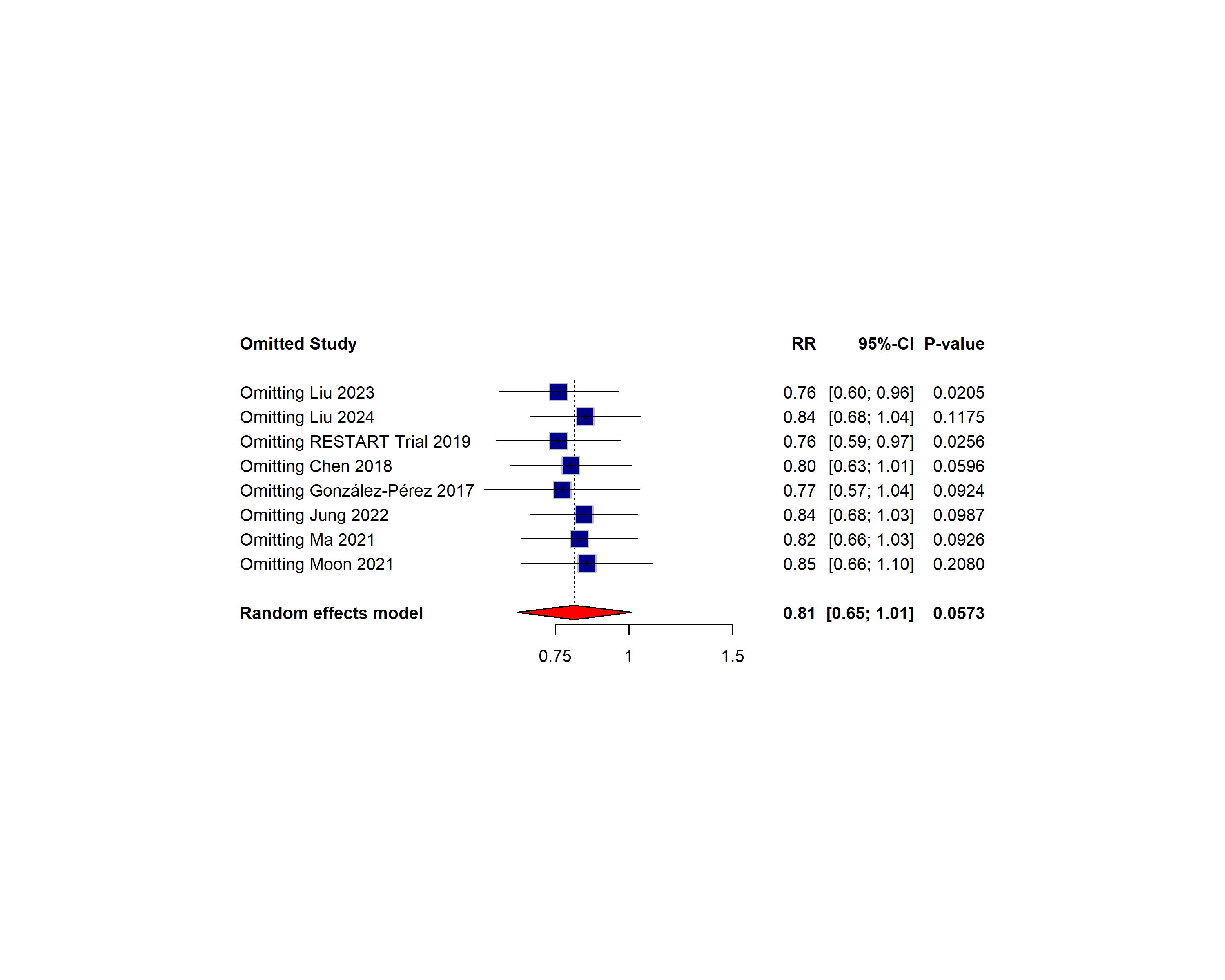


**Supplementary Figure 5. Galbraith Plot of All-Cause Mortality Outcome**

**
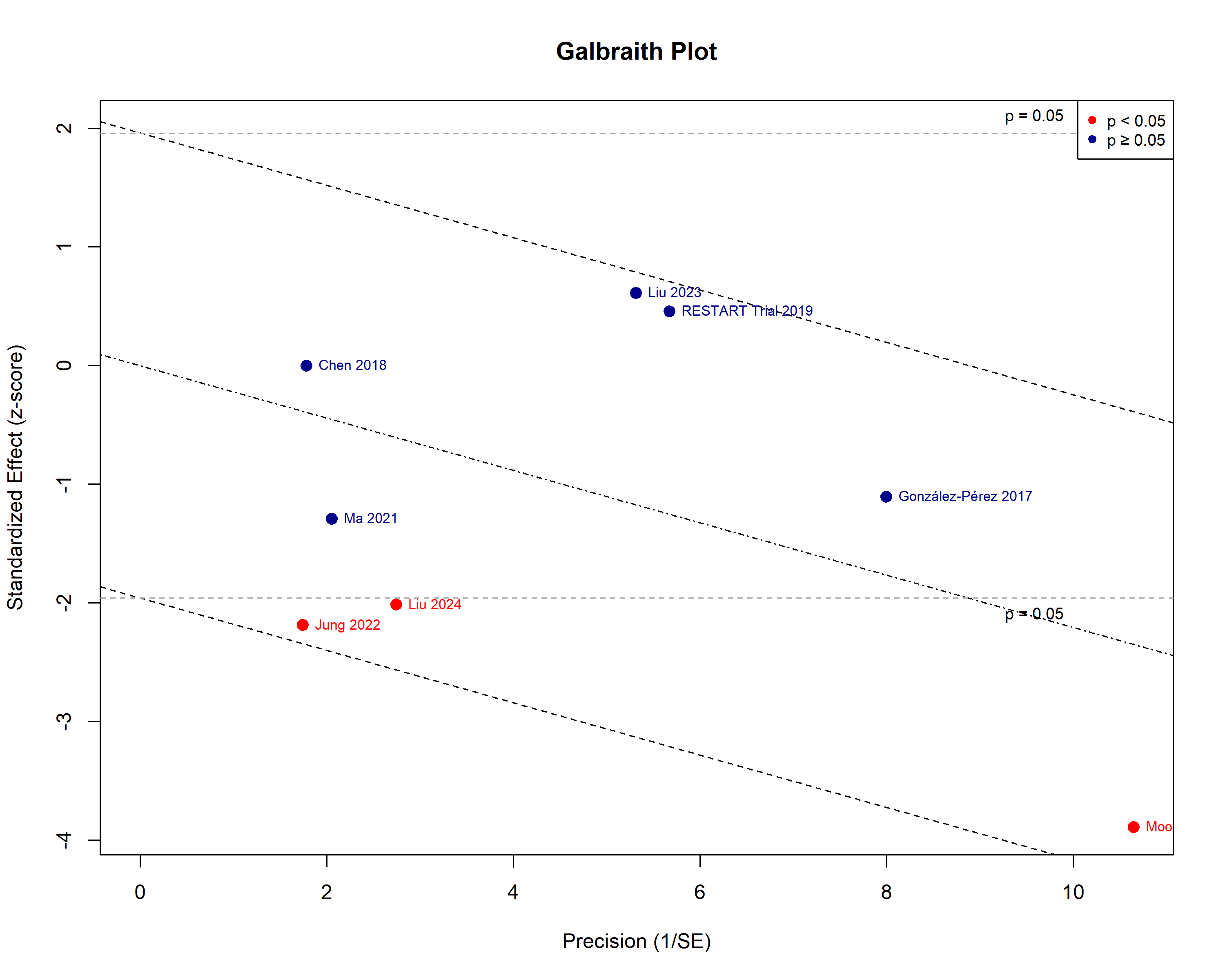
**
