# Supplementary material for: Antiplatelet Resumption After Intracerebral Hemorrhage: A Systematic Review and Meta-Analysis
Source: Diagnostics (Basel). 2025 Jul 15;15(14):1780. doi: 10.3390/diagnostics15141780 (PMC12293368; doi:10.3390/diagnostics15141780)
Supplement: Supplementary file 1 [file diagnostics-15-01780-s001.zip › Supplementary Table 2.docx]

**Supplementary Table 2. Baseline Characteristics and Summary of the Included Studies**

| **Study ID** | **Arms** | **Sample Size** | **Study Design** | **Country** | **Follow Up** | **ICH Location** | **Type of intervention/exposure** | **Dosage and duration** | **Timing of intervention** | **Age, mean (SD)** | **Male, n (%)** | **DM. n (%)** | **HTN, n (%)** | **Smoking, n (%)** | **Coronary artery disease, n (%)** | **Previous stroke/transient ischemic attack, n (%)** | **Atrial fibrillation, n (%** | **Intracerebral hemorrhage, n (%)** | **Subarachnoid hemorrhage, n (%)** | **Subdural hemorrhage, n (%)** | **HTN medication, n (%)** | **inclusion criteria** | **conclusion** |
| --- | --- | --- | --- | --- | --- | --- | --- | --- | --- | --- | --- | --- | --- | --- | --- | --- | --- | --- | --- | --- | --- | --- | --- |
| Chong 2011 | Aspirin Group | 56 | Single-center observational study | China | 62.2m | *Intracerebral Hemorrhage (ICH): 93.6% of all ICHs. 1-Lobar Hemorrhage: 34.4% of ICH cases. 2-Deep Cerebral Hemorrhage: 65.6% of ICH cases. *Subarachnoid Hemorrhage (SAH): 5.2%. *Subdural Hemorrhage (SDH): 1.2%. | Aspirin | 100 mg | NA | 64.2 (1.7 ) | 38 (67.8) | 16 (28.6) | 38 (67.9) | 19 (33.9) | 18 (32.1) | 11 (19.6) | 9 (16.1) | 52 (92.9) | 4 (7.1) | 0 (0) | NA | Consecutive Chinese patients with a first spontaneous ICH admitted to Queen Mary Hospital from May 1996 to February 2010. | We observed in a cohort of Chinese post-ICH patients that aspirin use was not associated with an increased risk for a recurrent ICH. |
|  | No Aspirin Group | 384 |  |  |  |  |  |  | NA | 58.5 (0.8) | 236 (61.5) | 53 (13.8) | 191 (49.7) | 97 (25.3) | 11 (2.9) | 28 (7.3) | 11 (2.9) | 360 (93.8) | 19 (4.9) | 5 (1.3) | NA |  |  |
| Flynn 2010 | Antiplatelet Group | 120 | Observational cohort study | Scotland | 36.5 m | *Lobar Hemorrhage: 235 patients (56.4%). *Deep Hemorrhage: 139 patients (33.3%). *Unclassified: 43 patients (10.3%). | Antiplatelet medicines (aspirin, clopidogrel, or dipyridamole) | NA | *18.3% of patients started antiplatelet therapy within 1 month. *24.2% started within 3 months. *46.7% started within 1 year | 70.3 | 58 (48) | 20 (16.7) | NA | NA | NA | 4 (3.3) | NA | NA | NA | NA | 22 (18.3) | Older than 18 years identified as having had a first ICH during the study period were included. | Antiplatelet prescribing was common after ICH. Subsequent ischemic strokes or myocardial infarctions were more common than recurrent ICH. Antiplatelet prescribing did not appear to have a clinically significant impact on outcomes measured. Despite being contraindicated, antiplatelet use was not a major hazard for recurrent ICH. |
|  | No Antiplatelet Group | 297 |  |  |  |  |  |  |  | 69.7 | 151 (50.8) | 41 (13.8) | NA | NA | NA | 12 (4) | NA | NA | NA | NA | 22 (18.3) |  |  |
| Liu 2023 | Early anti platelet Group | 843 | Retrospective cohort study | Taiwan | 1y | NA | NA | NA | within 30 days after ICH | 71.28 (11.97) | 547 (64.89) | 395 (46.86) | 668 (79.24) | NA | 681 (80.78) | NA | NA | NA | NA | NA | 723 (85.77) | The ICH patients were recruited from the National Health Insurance Research Database (NHIRD) released by Health and Welfare Data Science Center in Taiwan. | Early resumption of antiplatelet was as safe as delayed antiplatelet resumption in ICH patients. Besides, those without prior cerebrovascular disease or with chronic kidney disease may benefit more from early antiplatelet resumption. |
|  | Late anti platelet Group | 741 |  |  |  |  |  |  | between 31–365 days after ICH | 70.06 (11.71) | 475 (64.10) | 327 (44.13) | 602 (81.24) | NA | 619 (83.54) | NA | NA | NA | NA | NA | 637 (85.96) |  |  |
| Liu 2024 | Early anti platelet Group | 134 | Prospective, multicentre, open-label, blinded-endpoint, randomised trial | China | 3 m | *Supratentorial and deep: 170 (63%) patients. *Supratentorial and lobar: 72 (27%) patients. *Infratentorial: 27 (10%) patients. | Acetylsalicylic acid (aspirin) | 100 mg | on the third day after surgery and continued until the 90th day | 58.67 (11.99) | 96 (72) | 20 (15) | 119 (89) | 35 (26) | 14 (10) | 87 (65) | NA | NA | NA | NA | NA | Age 18–70 years, Non-traumatic, spontaneous intracerebral haemorrhage, Underwent neurosurgical procedure for haematoma evacuation (craniotomy, craniectomy, or minimally invasive surgery), High risk of ischaemic events (history of cerebral infarction, transient ischaemic attack, coronary heart disease, myocardial infarction, or a 10-year atherosclerotic cardiovascular disease risk score ≥10%), No history of allergy to salicylic acid preparations, Postoperative Caprini risk score > 2 (high risk of venous thromboembolism). | Starting acetylsalicylic acid on the third day after surgery for spontaneous intracerebral haemorrhage resulted in fewer postoperative ischaemic major cardiovascular, cerebrovascular, or peripheral vascular events than starting acetylsalicylic acid therapy at 30 days, with no increased risk of intracranial bleeding. Whether early initiation of acetylsalicylic acid therapy is safe and improves clinical outcomes. |
|  | Late anti platelet Group | 135 |  |  |  |  |  |  | on the 30th day after surgery and continued until the 90th day | 59.33 (10.5) | 99 (73) | 28 (21) | 124 (92) | 41 (30) | 12 (9) | 91 (67) | NA | NA | NA | NA | NA |  |  |
| RESTART Trial 2019 | Antiplatelet Group | 268 | Prospective, randomized, open label, blind endpoint, parallel-group trial | UK | 24 m | *Lobar Supratentorial: 62% of participants. *non-lobar: 38% of participants. | NA | NA | NA | 76 (9.69) | 173 (65) | NA | NA | NA | NA | 57 (21) | 42 (16) | NA | NA | NA | NA | Adults (≥18 years) who survived at least 24 hours after spontaneous intracerebral haemorrhage confirmed by brain imaging, Participants were taking antithrombotic (antiplatelet or anticoagulant) therapy for the prevention of occlusive vascular disease at the onset of intracerebral haemorrhage, which was subsequently discontinued, Participants or their nearest relative/representative provided written informed consent. | The risk of recurrent intracerebral haemorrhage is probably too small to exceed the established benefits of antiplatelet therapy for secondary prevention. |
|  | No Antiplatelet Group | 269 |  |  |  |  |  |  |  | 75.67 (9.69) | 187 (70) | NA | NA | NA | NA | 76 (28) | 50 (19) | NA | NA | NA | NA |  |  |
| Chen 2018 | Antiplatelet Group | 107 | Multicenter, retrospective matched cohort study | US | 3 m | *Lobar: 29.6% in APT group, 32.5% in no APT group. *Deep: Not explicitly broken down in the paper. *Infratentorial: 13.6% in APT group, 11.4% in no APT group. | NA | NA | NA | 65.3(11.1) | 72 (67.3) | 47 (43.9) | 97 (90.7) | 27 (42.9) | 35 (32.7) | 28 (26.2) | 19 (17.8) | NA | NA | NA | NA | Age ≥18 years,Patients taking APT before presentation with ICH, Alive at the time of hospital discharge. | Restarting APT in patients with ICH of mild to moderate severity after acute hospitalization is not associated with worse functional outcomes or health-related quality of life at 90 days. In patients with significant cardiovascular risk factors who experience an ICH, restarting APT remains the decision of the treating practitioner. |
|  | No Antiplatelet Group | 107 |  |  |  |  |  |  |  | 65.6(11.8) | 65 (60.8) | 48 (44.9) | 101 (94.4) | 19 (28.4) | 31 (29.0) | 37 (34.6) | 17 (15.9) | NA | NA | NA | NA |  |  |
| González-Pérez 2017 | Aspirin Group | 286 | Retrospective longitudinal follow-up study using the Health Improvement Network (THIN) database. | UK | 6.4 y | NA | Low-dose aspirin | 75 to 300 mg/day | 30 days after the hemorrhagic stroke | NA | 1,016.2 (49) | 221.2 (10.7) | 1,142.1 (55) | 602.2 (29) | NA | NA | NA | 1,105.2 (53.3) | 970.2 (46.7) | NA | NA | Patients who survived at least 30 days after a first non-traumatic haemorrhagic stroke (HS) event between 2000 and 2008 | Our results suggest an improved survival associated with low-dose aspirin. This finding must be interpreted with care due to the observational nature of the study, and warrants further studies. |
|  | No Aspirin Group | 286 |  |  |  |  |  |  |  | NA | 3,868.4 (43.1) | 504.1 (5.6) | 3,114.1 (34.7) | 2,556.4 (28.5) | NA | NA | NA | 3,922.9 (43.7) | 5,046.4 (56.3) | NA | NA |  |  |
| Jung 2022 | Antiplatelet Group | 118 | Retrospective observational study | Korea | 43 m | NA | Single APT (e.g., aspirin)  dual APT (e.g., aspirin + clopidogrel). | NA | 6 months after recovering from the initial ICH | 66.7 (10.6) | 56 (47.5) | 31 (26.3) | 94 (79.7) | NA | NA | 72 (61) | 12 (10.2) | NA | NA | NA | NA | Adult patients (aged ≥ 18 years) who regularly took APT medications for various medical conditions and who had survived after acute hospitalization for initial ICH events. Spontaneous ICH was diagnosed based on the neurologic condition and neuro-radiolgical findings on brain computed tomogryphy or magnetic resonance imaging (MRI) scans. | Our findings indicated that restarting APT in patients with ICH was not associated with an increased risk of recurrent ICH. APT can be safely restarted to prevent major thromboembolic complications in patients on previous antithrombotic treatment. |
|  | No Antiplatelet Group | 84 |  |  |  |  |  |  |  | 68.7 (10.5) | 44 (52.4) | 22 (26.2) | 64 (76.2) | NA | NA | 7 (8.3) | 7 (8.3) | NA | NA | NA | NA |  |  |
| Ma 2021 | Antiplatelet Group | 151 | Retrospective hospital-based study | China | 48 m | NA | *Aspirin *clopidogrel | *Aspirin (75 mg daily)  *clopidogrel (50 mg/day) | NA | 62 (10.90) | 96 (63.6) | 68 (45.03) | 98 (64.90) | 66 (49.71) | NA | NA | 38 (25.17) | NA | NA | NA | NA | Patients aged ≥18 years who survived for >90 days after ICH, Cranial CT confirmed the presence of ICH | Low-dose AT at a median of 6.2 months was associated with a lower risk of ischemic vascular events without increased risk of recurrent ICH. Low-dose AT after ICH may be an option for Chinese clinicians to balance the risk of ischemic vascular events and recurrent ICH. |
|  | No Antiplatelet Group | 161 |  |  |  |  |  |  |  | 65 (11.57) | 94 (54.8) | 77 (47.82) | 111 (68.94) | 71 (44.10) | NA | NA | 17 (10.56) | NA | NA | NA | NA |  |  |
| Moon 2021 | Antiplatelet Group | 363 | Retrospective cohort study | South Korea | 2.47 y | NA | Low-dose acetylsalicylic acid (aspirin), clopidogrel, cilostazol, ticagrelor, and ticlopidine. | NA | Restarted at a median of 0.96 years after ICH | NA | 532 (52.8) | 292 (29) | 805 (80) | NA | NA | NA | NA | NA | NA | NA | NA | Patients who survived an ICH event, Patients with emergency management charge codes, brain CT or MRI codes, and ICH codes (ICD-10: I61.x). | Using antiplatelet treatment after ICH does not increase chances of recurrence, but lowers the occurrence of subsequent clinical events, especially mortality. However, the prescription and resumption rate of anti-platelet therapy after ICH remains low in South Korea. |
|  | No Antiplatelet Group | 644 |  |  |  |  |  |  |  |  |  |  |  |  | NA |  |  |  |  |  |  |  |  |
| ***Abbreviations: ICH = Intracerebral Hemorrhage; SAH = Subarachnoid Hemorrhage; SDH = Subdural Hemorrhage; APT = Antiplatelet Therapy; HTN = Hypertension; DM = Diabetes Mellitus; CAD = Coronary Artery Disease; TIA = Transient Ischemic Attack; SD = Standard Deviation; NA = Not Available*** | | | | | | | | | | | | | | | | | | | | | | | |
