# Supplementary figures and images for: Antiplatelet Resumption After Intracerebral Hemorrhage: A Systematic Review and Meta-Analysis
Source: Diagnostics (Basel). 2025 Jul 15;15(14):1780. doi: 10.3390/diagnostics15141780 (PMC12293368; doi:10.3390/diagnostics15141780)

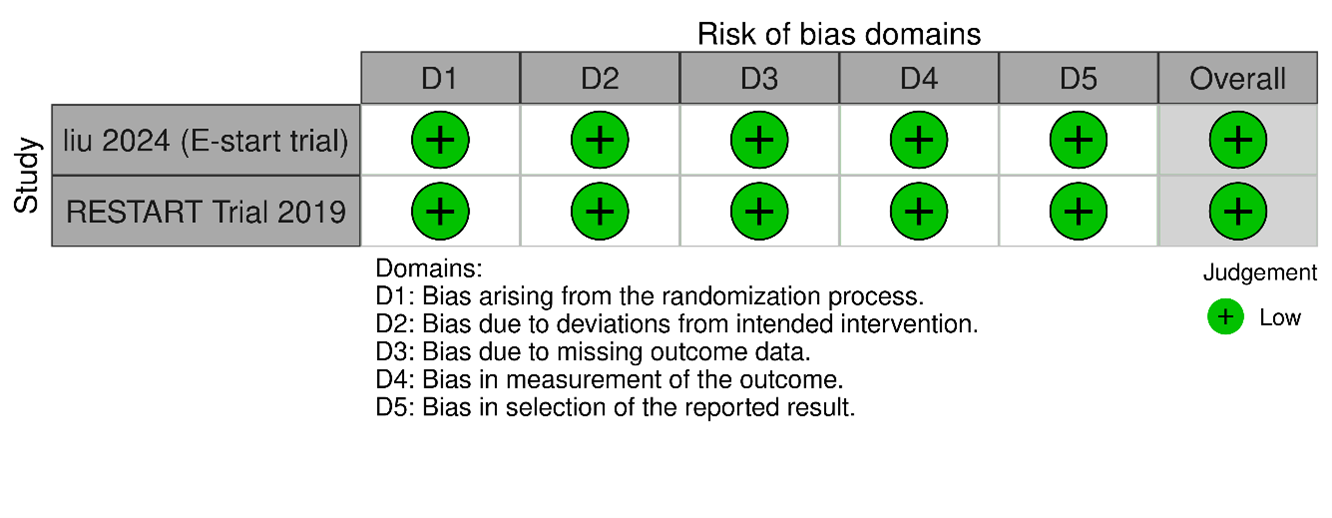

Supplement: Supplementary file 1 [file diagnostics-15-01780-s001.zip › Supplementary Figures/Supplementary Figure 1 Risk of bias assessment using ROB-2 tool.png]

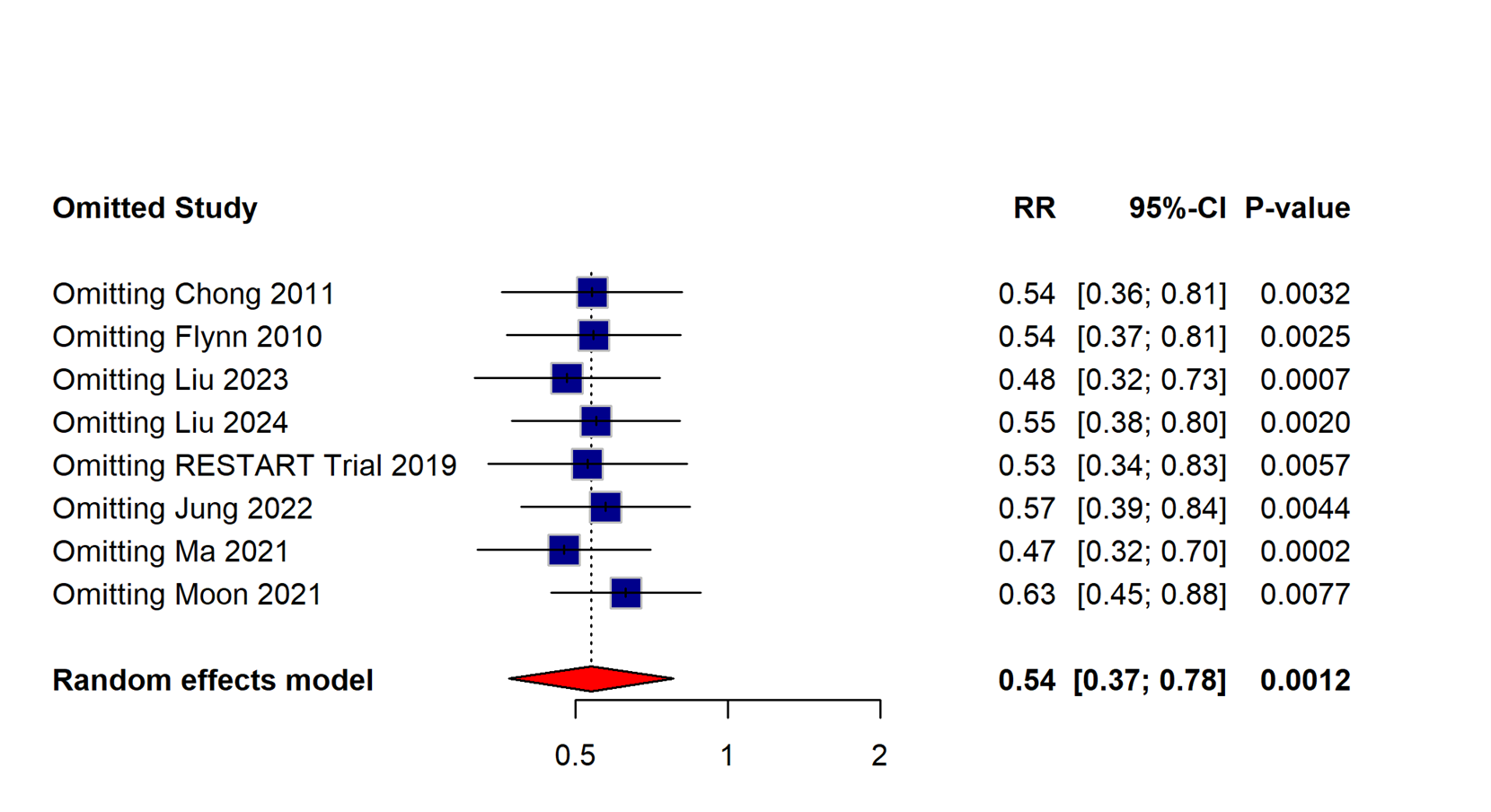

Supplement: Supplementary file 1 [file diagnostics-15-01780-s001.zip › Supplementary Figures/Supplementary Figure 2 Leave-One Out Analysis of Recurrent ICH Outcome.png]

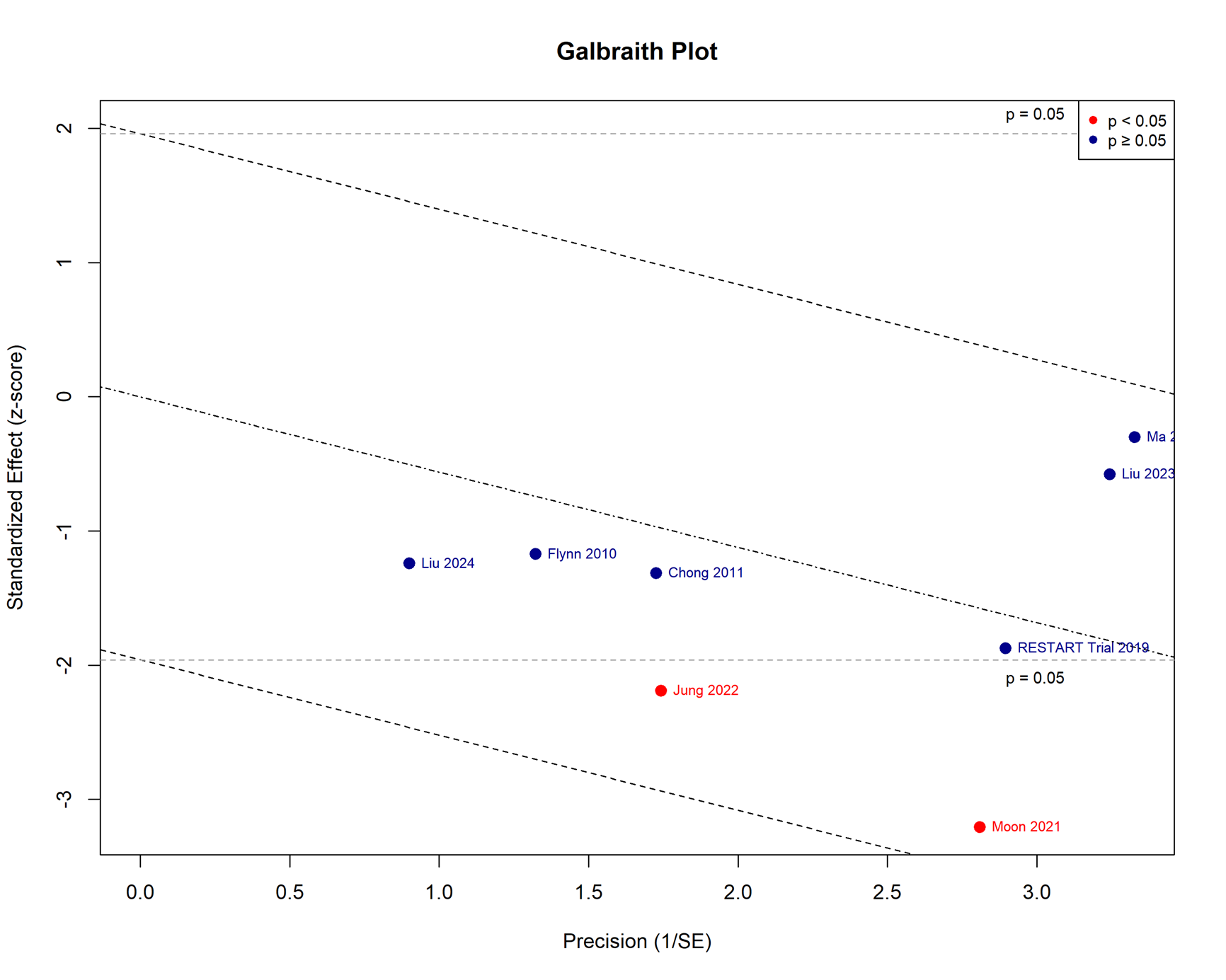

Supplement: Supplementary file 1 [file diagnostics-15-01780-s001.zip › Supplementary Figures/Supplementary Figure 3 Galbraith Plot of Recurrent ICH Outcome.png]

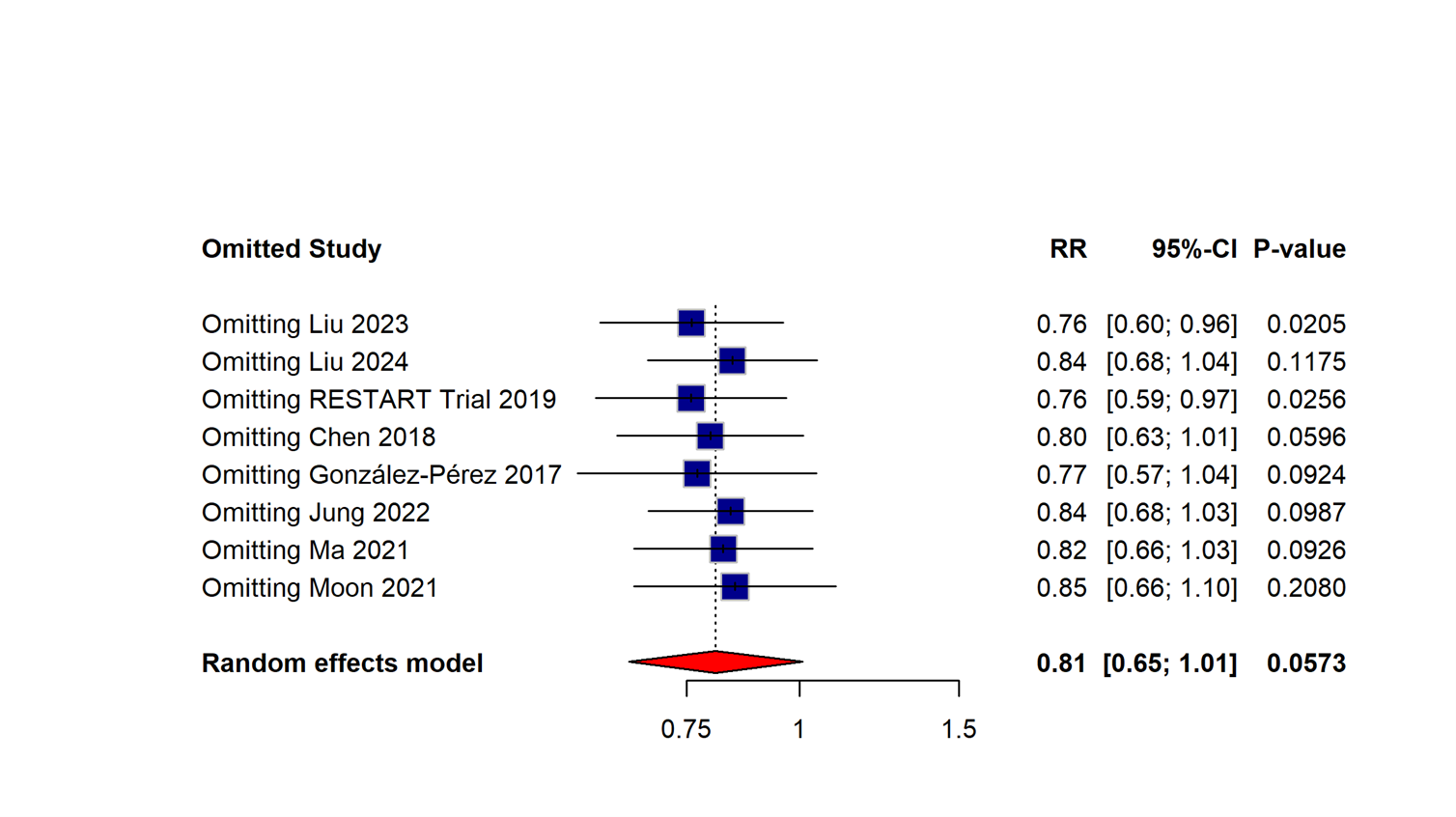

Supplement: Supplementary file 1 [file diagnostics-15-01780-s001.zip › Supplementary Figures/Supplementary Figure 4 Leave-One Out Analysis of All-Cause Mortality Outcome.png]

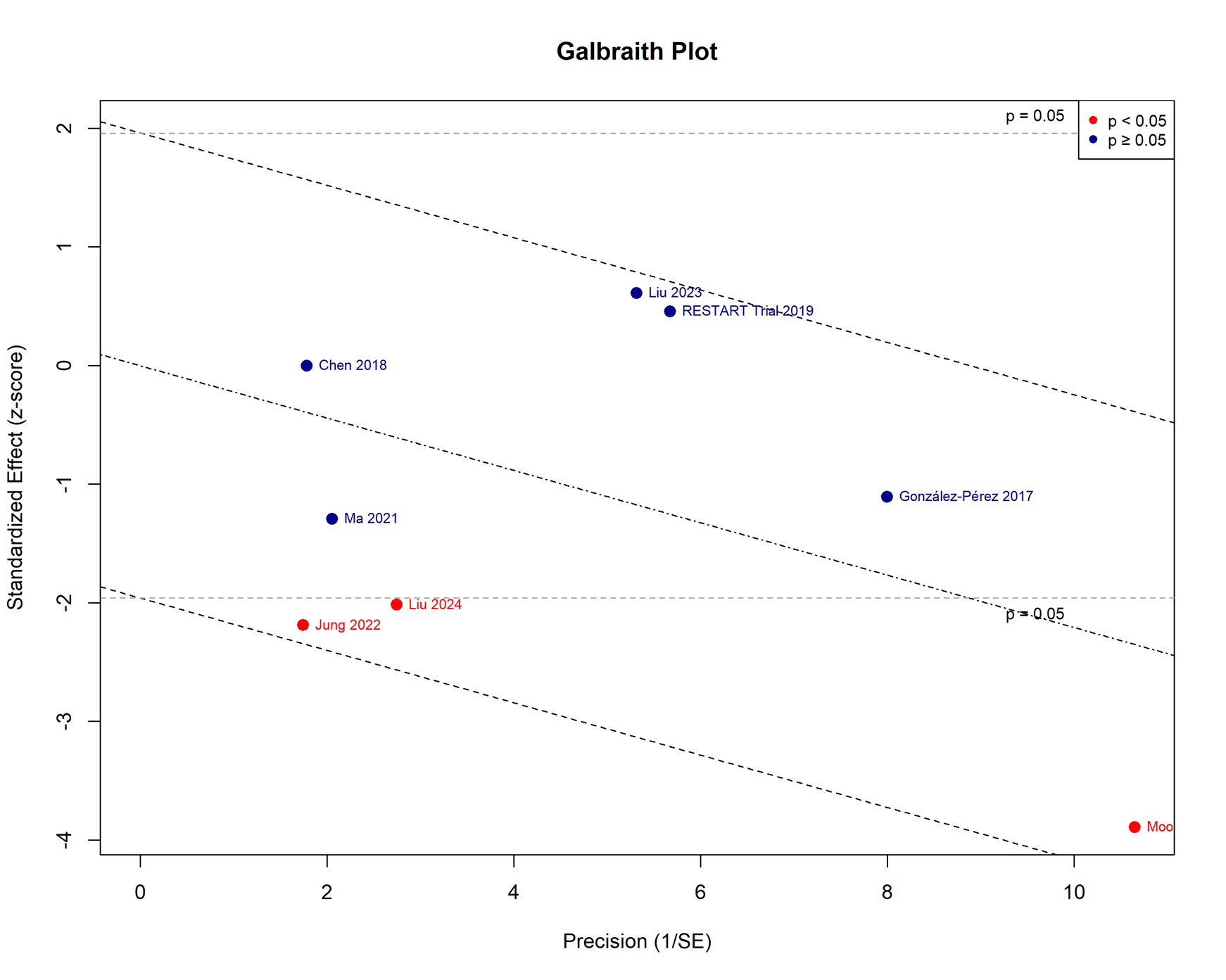

Supplement: Supplementary file 1 [file diagnostics-15-01780-s001.zip › Supplementary Figures/Supplementary Figure 5 Galbraith Plot of All-Cause Mortality Outcome.png]
